# Dynamic Recrystallization Critical Conditions and a Physically–Based Constitutive Model of Al–4.8Mg Alloy Under Hot Working Conditions

**DOI:** 10.3390/ma13214982

**Published:** 2020-11-05

**Authors:** Qingsong Dai, Yunlai Deng, Yu Wang, Wenhui Huang

**Affiliations:** 1 School of Materials Science and Engineering, Central South University, Changsha 410083, China; daiqingsong@alzco.com.cn (Q.D.); luckdeng@csu.edu.cn (Y.D.); 2 Guangxi Liuzhou Yinhai Aluminum Co., Ltd., Liuzhou 545006, China; hwh19860715@163.com

**Keywords:** Al–4.8Mg alloy, hot deformation behavior, dynamic recrystallization critical conditions, physically–based constitutive model

## Abstract

The microstructure evolution and the mechanical behavior of Al–4.8Mg alloy were investigated by means of isothermal compression tests at various temperatures (280–520 °C) and strain rates (0.01–10 s^−1^). The results shown that there are three main mechanisms of dynamic softening of samples within the range of selected process parameters: dynamic recovery, dynamic recovery + dynamic recrystallization, and dynamic recrystallization, and the equiaxed dynamic recrystallization grain tends to be formed under higher temperature and higher strain rate. In order to accurately describe the dynamic recrystallization condition of Al-4.8Mg alloy under a wide range of hot deformation parameters, an improved dynamic recrystallization critical conditions model is proposed based on deformation activation energy and work-hardening rate. Additionally, a two–stage physically constitutive model considering the influence of work hardening–dynamic recovery and dynamic recrystallization is established. Comparisons between the predicted and experimental data indicate that the proposed model can adequately predict the flow stress in the range of selected process parameters with the average absolute relative error of 4.02%.

## 1. Introduction

Due to their characteristics of light weight, reasonable strength, good formability and high corrosion resistance, Al-Mg alloys are widely used in transportation, architectural decoration, food packaging and other industrial fields [1,2,3]. Thermal processes such as rolling, extruding, and forging are the main Al-Mg alloy manufacturing methods. During the hot deformation process, complex microstructural evolutions, such as dislocation movement, dynamic recovery (DRV) and dynamic recrystallization (DRX) can take place [4,5]. The coupling effects of these microstructural evolutions make the thermal deformation behavior of alloys very complicated. Therefore, it is of great significance for process designing, equipment selection and properties controlling of the manufacturing of the alloys to study the exact mechanisms of the hot deformation behaviors.

Because of its high stacking fault energy, DRX of aluminum alloys is conventionally considered to be difficult to happen during hot deformation. However, with the in-depth research, DRX was proved to be an important softening mechanism during hot deformation of aluminum alloy. In recent years, the phenomena of DRX in different aluminum-based alloy systems were successively reported by many works, such as Al-Cu [6], Al-Li [7], Al-Zn-Mg [8], Al-Mg-Si [9] and Al-Mg-Mn [10]. In these various works, the corresponding thermo-mechanical conditions of the DRX have been revealed. Accordingly, phenomenological constitutive models have been proposed to describe the rheological behaviors of the alloys during hot deformation based on the correlations between experimental conditions and stress-strain behaviors. The frequently-used phenomenological constitutive models include Jonnson–Cook (J–C) model, Zerilli−Armstrong (Z-A) model and Arrhenius–type with Zener−Hollomon parameters (Z−H) et, al. These models have been extensively used to describe the hot deformation behaviors of numerous alloys, such as 6062 [11], 7050 [12], 35CrMo steel [13], AZ41M magnesium alloy [14], V-5Cr-5Ti [15] alloy and Cu-Cr-Zr-Y alloy [16]. However, the mentioned phenomenological constitutive models cannot profoundly reflect the deformation mechanisms and the microstructural evolutions of the alloys. Therefore, physically–based constitutive model based on classical dislocation theory and Avrami kinetics were developed and were applied to explain the deformation behaviors of Al-Zn-Mg-Cu alloys [17], Al-Li alloy [18], Cu-0.4Mg alloy [19] and nickel-based superalloy et al. [20].

Generally, the thermal mechanical conditions for DRX of aluminum alloy are more severe than those for DRV. Therefore, there are should be a series of critical parameters from DRV to DRX. In the whole range of selected process parameters, if no DRX occurred in the material, the physical constitution based on DRV was used to describe the deformation behavior of the material. Such as, the deformation behavior of 5754 aluminum alloy reported by Huang [21]. Moreover, when DRX occurs in the whole range of selected process parameters, as reported in Al-Li alloy [22], the inflection point of θ–σ curve is used to obtain the critical strain of DRX (θ is the work hardening rate). This inflection point was generally treated as the critical conditions of DRX. These works used the critical strain condition to accurately reflect the physical characteristics of DRV or DRX in the process of deformation. However, the critical strain condition of DRX is obviously not suitable for the case of partial DRX within a wide range of process parameters. Actually, the influence of temperature and strain rate on DRX is more significant than the strain. When the deformation temperature and strain rate cannot reach the condition of DRX, no matter how large the strain is, DRX is difficult to occur. Therefore, it is necessary to establish a critical conditions model of DRX based on the coupling of hot deformation temperature, strain rate and strain rate for Al-Mg alloys with a wide range of temperature and strain rate parameters.

In order to accurately describe the DRX conditions of Al‒4.8Mg alloy under a wide range of hot deformation parameters, in this paper, the hot deformation behavior of Al‒4.8Mg alloy was investigated via a set of hot compression tests at temperatures of 280‒520 °C and strain rates of 0.01‒10 s^−1^. An improved DRX critical conditions model of Al–4.8Mg alloy is proposed based on deformation activation energy and work-hardening rate. Further, a physically‒based model considering DRV and DRX mechanisms is established to describe the relationship between the flow stress and forming parameters.

## 2. Materials and Experiments

**Figure 1:**
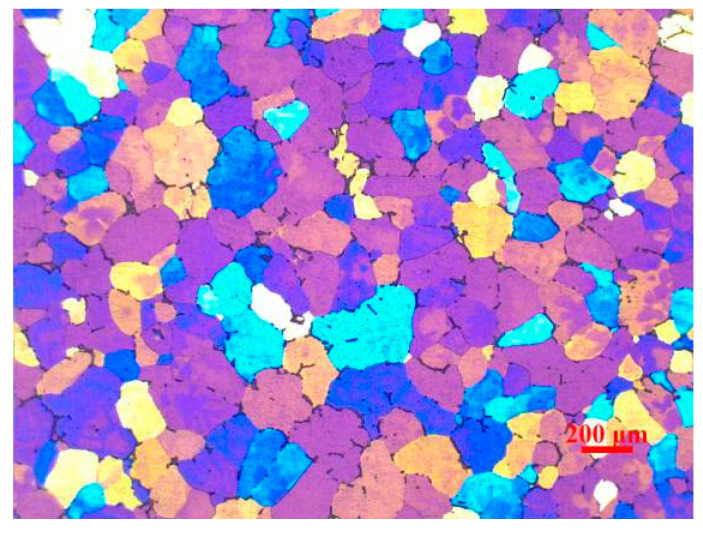
Original optical microstructure of the Al–4.8Mg alloy.

The experimental material employed in this paper is prepared by the industrial production line. It is cast into flat ingots after strict degassing and deslagging in the industrial process, and then the flat ingots were homogenized by 420 °C/2 h + 500 °C/12 h. The chemical composition (wt.%) of Al-Mg alloy (Guangxi Liuzhou Yinhai Aluminum Co., Ltd, Liuzhou, China) is 4.82Mg–0.49Mn–0.11Cr–0.29Fe–0.11Si–(Bal.) Al. The optical microstructure of the initial homogenized ingot exhibits uniform equiaxed grains, with an mean grain size of 180 um, as shown in Figure 1.

The homogenized ingot was machined to cylindrical specimens with 15 mm (height) × 10 mm (diameter) in size. The compression tests were carried out on a Gleeble–3500 thermal simulator (Data Science International, INC, St. Paul, MN, USA) at temperatures from 280~520 °C, strain rates of 0.01~10 s^–1^ and strain of ~0.9. Prior to hot compression, all specimens were heated up to the target compression temperatures at a heating rate of 10 °C/s and held at the testing temperature for 3 min to obtain a uniform and stable temperature. After each compression test, all samples were quenched in cold water quickly to maintain the deformation microstructure.

The compressed specimens were cut along the deformation axis for the microstructural observations. The microstructural characterizations were conducted on an AXIOScope A1 optical microscope (OM, Carl Zeiss, Munich, Germany), an electron back-scattered diffraction (EBSD) and a TecnaiG2 20 transmission electron microscopy (TEM, FEI, Hillsboro, OR, USA). The OM samples were polished and anode–coated at a voltage of 20 V for 1 min using a solution of fluoroboric acid (10 mL) + water (400 mL). The EBSD samples were electrolytically polished at 20 V for 5 s in 10% HCHO_4_ + 90% C_2_H_5_OH solution and then observed using a Sirion 200 field-emission gun SEM (Hillsboro, OR, USA) at 25 kV with a typical scan step size (1.5 µm). The TEM samples were first mechanically grinded and polished to less than 100 μm, then punched into discs with a diameter of 3 mm and finally twin–jet electropolished with an electrolyte solution of 30% nitric acid and 70% methanol at a voltage of ~15 V DC and a temperature below −25 °C.

## 3. Results and Discussion

### 3.1. True Stress–Strain Curves

**Figure 2:**
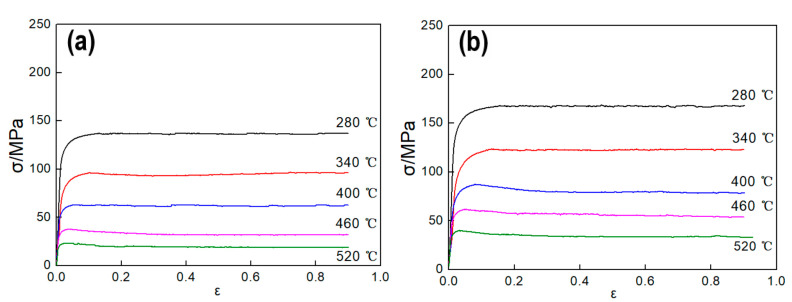

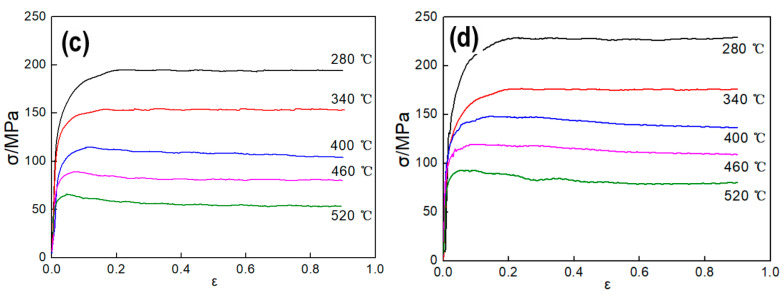
The true stress–strain curves under various strain rates: (**a**) 0.01 s^−1^, (**b**) 0.1 s^−1^, (**c**) 1 s^−1^, (**d**) 10 s^−1^.

Figure 2 show the true stress–strain curves of the experimental samples tested at various hot deformation conditions. Obviously, the flow stress is closely related to the temperature and strain rate. The flow stress decreases with the increasing of the temperature at the given strain rate, while increases with the increasing of the strain rate at the given temperature. This variation tendency of flow stress can be explained in terms of work hardening (WH) and dynamic softening (DRV and DRX) [23,24,25]. As shown in Figure 2, all the true stress–strain curves can be divided into three stages in features, namely, sharply rising stage, slowly rising stage and steadily stage. First, in the sharply rising stage, dislocation density in the sample increases rapidly under the effects of the applied stress, which results in obvious WH. Therefore, the true stress in this stage increases rapidly. Second, with continuing of deformation, dislocation density increases, and the deformation storage energy in the sample accumulates continually, which provides driving force to the DRV and DRX and results in softening. 

When the softening rate and work hardening rate reach an equilibrium condition, the flow stress attains the maximum, i.e., the peak stress. In the steadily stage, the stress–strain curves show two change characteristics. If there only DRV occurs when the stress exceeds the peak strain, the flow stress tends to be stable after the dynamic equilibrium of WH and dynamic softening is achieved. If the dynamic softening rate caused by DRX is greater than the hardening rate, the flow stress will gradually decrease until the deformation reaches a certain strain point, the WH and dynamic softening reach a new equilibrium point, and the flow stress tends to be stable.

### 3.2. Microstructural Evolution

Table 1 displays the optical microstructures of Al-4.8Mg alloy samples under various deformation conditions. The initial coarse grains were elongated to fiber texture at higher strain rate and fine dynamic recrystallization grains occurred at higher temperature. There are three main mechanisms of dynamic softening of samples within the range of selected process parameters: DRV, DRV + DRX and DRX, as shown in Table 1. For example, no DRX occurred in the sample at 400 °C and strain range of 0.01 s^−1^, which represents the softening mechanism dominated by DRV. When the deformation temperature ranges from 400 °C to 460 °C and the strain rate ranges from 0 to 1 s^–1^, fine DRX grains and large numbers of sub-grains can be observed from the micrographs, as shown in Table 1, which indicates DRX has occurred, i, e. the dominating softening mechanism is DRV+DRX. Additionally, large numbers of DRX grains can be found in the micrograph of the 520 °C/10 s^–1^ samples. This means the softening mechanism of this sample is dominated by DRX.

**Figure 3:**
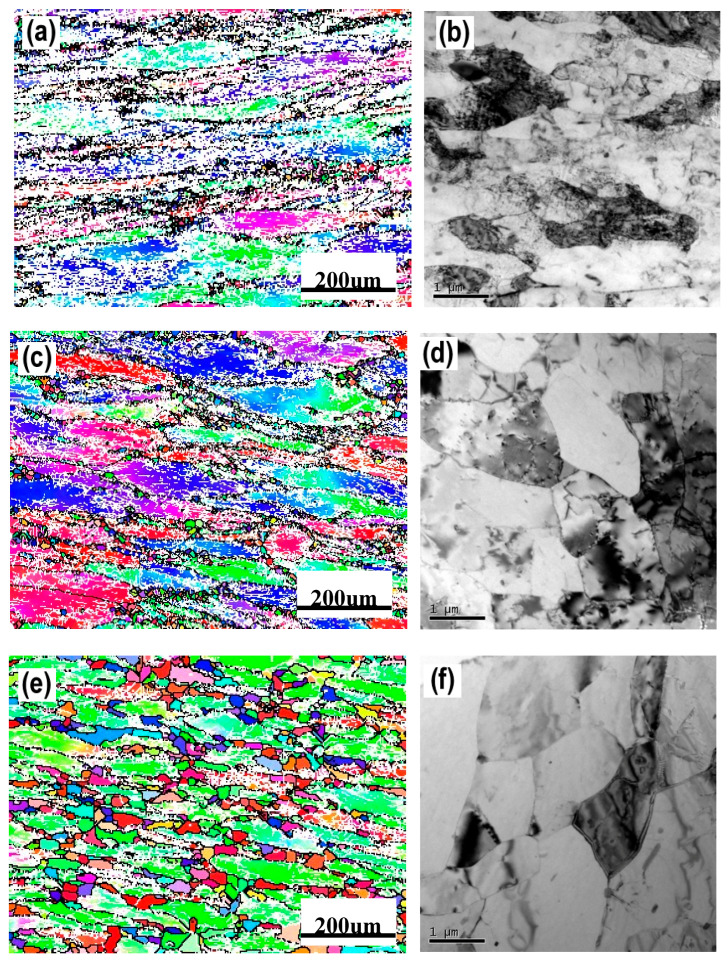
The microstructure of Al–4.8Mg alloy at various deformation conditions: (**a**), (**b**) *T* = 280 °C, $\dot{\varepsilon }$ =1 s^–1^; (**c**), (**d**) *T* = 400 °C, $\dot{\varepsilon }$ = 1 s^–1^; (**e**), (**f**) *T* = 520 °C, $\dot{\varepsilon }$ = 1 s^–1^.

Figure 3 shows the typical microstructures of the samples under different dominating softening mechanisms. On the EBSD and TEM visual fields, the sample with deformation parameters of 280℃/1s^−1^, which dominated by DRV softening mechanism, represents large numbers of dislocations and almost no DRX grains, as shown in Figure 3a,b. When the deformation temperature increased to 400 °C, DRX grains can be obviously observed by EBSD, and intragranular dislocations can be easily found on the corresponding TEM micrograph, which further confirms the DRV+DRX jointly dominated softening, as Figure 3c,d shows. When the deformation condition is 520 °C /1 s^−1^, there are many DRX grains in the sample and the recrystallization degree is obviously higher than that of the sample deformed at 400 °C /1 s^−1^. According to the observation results of EBSD and TEM, Figure 3e,f, there are more fine recrystallized grains and fewer dislocations in the sample. This indicates that the softening of the sample deformed at 520 °C /1 s^−1^ is dominated by DRX. These results are consistent with the optical microstructures as shown in Table 1.

According to the microstructural characterizations, it can be inferred that the samples deformed at different conditions represent different softening mechanisms. Obviously, the variation of softening mechanisms with process parameters must be fully considered, when establishing the physical constitutive model. For instance, in this paper, in some conditions, the main softening mechanism of the experimental samples is DRV, while in some conditions the softening mechanism is dominated by DRV and DRX. Therefore, there must be a critical condition for DRX of the samples, which should be considered. However, the critical strain of DRX determined by the inflection point of the θ–σ curve proposed in [22] is only applicable to the case that DRV and DRX jointly dominate the softening behavior under all the experimental processing conditions. This is obviously not suitable for establishing the physical-based constitutive model of Al-Mg alloy in this paper. Therefore, it is necessary to study the critical conditions of DRX based on the coupling of hot deformation temperature, strain rate and strain in detail.

### 3.3. DRX Critical Conditions

According to the microstructure characterizations, only when the temperature and strain rate meet a certain condition DRX in the experimental samples can occur. Whereas the temperature and strain rate are suitable for DRX, DRX occurs only after the strain achieves a critical value [22]. Therefore, in order to ascertain the critical conditions of DRX, the critical temperature and strain rate should be determined first, and then the critical strain should be determined within this range.

#### 3.3.1. Determination of Critical Temperature and Strain Rate 

Generally, Zener–Hollomon parameter can be considered as a criterion of DRX. Many researchers [23,24] reported that the lower lnZ is, the easier the occurrence of DRX is. For determining lnZ, the logarithmic equation for describing the relationship between lnZ and the deformation parameters was adopted, as shown in Equation (1) [10]:

(1) $$\ln Z=\ln\dot{\varepsilon }+Q/RT$$

where, $\dot{\varepsilon }$ is the strain rate (S^−1^), *T* is the temperature (K), *Q* denotes the activation energy (J mol^−1^), and *R* is a constant (8.314 J mol^−1^ K^−1^). In order to get lnZ under different deformation conditions, the Arrhenius constitutive equation was employed, as described by Equation (2) [26]:

(2) $$\dot{\varepsilon }=Af(\sigma )\exp[-Q/(RT)]$$

where [26]:

(3) $$f\left(\sigma \right)=\{\begin{array}{l} \;\;\;\sigma^{n_{1}}\alpha \sigma <0.8\;\\ \;\exp\left(\beta \sigma \right)\;\;\alpha \sigma >1.2\\ \sinh{\left(\alpha \sigma \right)}^{n}\;\;\;for\;\;\;all\;\;\sigma \; \end{array}$$

Therein, σ is the flow stress (MPa), *n_1_*, *β, α*, *n* are material constants and *α = β/n_1_*. Substituting Equation (3) into Equation (2), then taking the natural logarithms of both sides, then Equations (4)–(6) are obtained:

(4) $$\ln\dot{\varepsilon }=\ln A_{1}-Q/RT+n_{1}\ln\sigma$$

(5) $$\ln\dot{\varepsilon }=\ln A_{2}-Q/RT+\beta \sigma$$

(6) $$\ln\dot{\varepsilon }=\ln A-Q/RT+n\ln[\sinh(\alpha \sigma )]$$

By transforming the Equations (4)–(6) and taking partial differentiation, the following expression are obtained:

(7) $$n_{1}={\left(\frac{\partial \ln\dot{\varepsilon }}{\partial \ln\sigma }\right)}_{T}$$

(8) $$\beta ={\left(\frac{\partial \ln\dot{\varepsilon }}{\partial \sigma }\right)}_{T}$$

(9) $$n={\left(\frac{\partial \ln\dot{\varepsilon }}{\partial \ln[\sinh(\alpha \sigma )]}\right)}_{T}$$

(10) $$Q=R{\left\{\frac{\partial \ln[\sinh(\alpha \sigma )]}{\partial \left(1/T\right)}\right\}}_{\dot{\varepsilon }}{\left\{\frac{\partial \ln\dot{\varepsilon }}{\partial \ln[\sinh(\alpha \sigma )]}\right\}}_{T}$$

**Figure 4:**
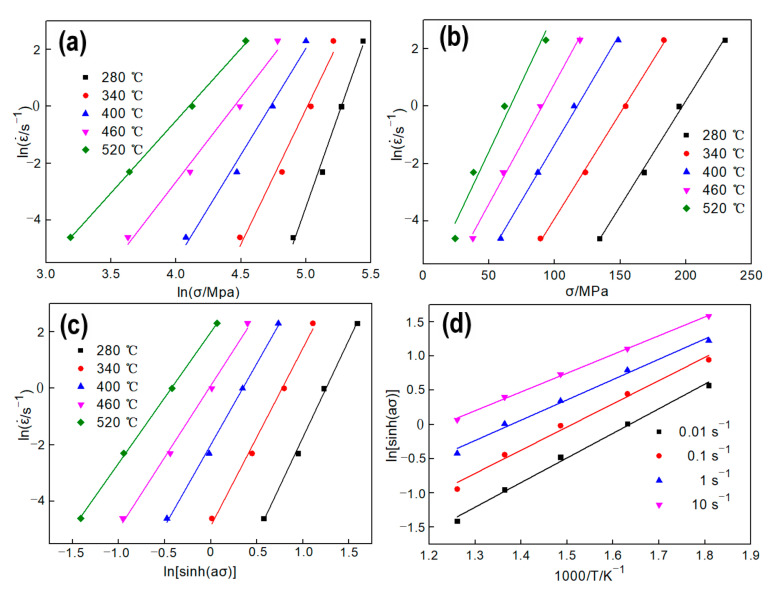
Relationship between peak stress and deformation conditions. (**a**) $\ln\sigma -\ln\dot{\varepsilon }$, (**b**) $\sigma -\ln\dot{\varepsilon }$, (**c**) $\ln[\sinh(\alpha \sigma )]-\ln\dot{\varepsilon }$, (**d**) ln[sinh(ασ)]−1/T.

Substituting the peak flow stress data at various deformation conditions into Equations (7) and (8), as shown in Figure 4a,b, $n_{1}$ and *β* can be calculated through the mean slopes by plotting $\ln\sigma -\ln\dot{\varepsilon }$ and $\sigma -\ln\dot{\varepsilon }$ under difference deformation temperatures, respectively. Thus, $n_{1}$ = 8.197, *β =* 0.0817, and *α* = *β/*$n_{1}$ = 0.00997. similarly, *n* and *S* can be obtained from Figure 4c and Figure 4d, respectively. Thus, *n* = 3.4519 and *S* = 6.0886. Substituting the above material coefficients into Equation (10), the average value of *Q =* 174.7 KJ/mol can be got, which is close to the result (168.61 KJ/mol) of Ref. [10]. As reported in Refs. [20,22], the average Q is usually employed into Equation (3) to calculate the lnZ at different deformation conditions.

Table 2 shows the lnZ of the experimental samples in each condition of this paper. It can be found in Table 2 that the value of lnZ decreases with the increasing of deformation temperature and increases with the increasing of strain rate. According to Table 1 and Table 2, it is not the lower lnZ is, the easier the occurrence of DRX is. For example, DRX occurs in the samples under deformation condition of 340 °C/10s^−1^, but no DRX occurs in the 400 °C/0.01 s^−1^, and their lnZ equals to 36.6 and 26.6, respectively. These results are inconsistent with results of 99.99% polycrystalline aluminum reported by [24]. In [24], there is an obvious relationship between Z parameter and DRX, DRX takes place when lnZ < 28, and only DRV takes place when lnZ > 28.

It is generally believed that increasing temperature is beneficial to atom migration and dislocation movement, whereas increasing strain rate is beneficial to energy accumulation in the samples during hot deformation processing. The coupling effects of the deformation temperature and strain rate is complex. When describing the critical conditions of DRX, Z parameter reflects the sum of the contribution of strain rate and deformation temperature to DRX, however, it is a general relationship, and the weighting factors of deformation temperature and strain rate that contribute to DRX are not considered. Additionally, lnZ in Table 2 was calculated using the average Q of the whole experimental range, which affected the accuracy of calculation. Therefore, in a wide range of process parameters, due to the complexity of the coupling effects between strain rate and deformation temperature, it is not appropriate to use the value of lnZ in Table 2 to describe the critical conditions of DRX.

Generally, deformation activation energy *Q* is an important physical parameter for hot deformation processing. The higher the *Q* is, the more difficult of the sample is to be deformed, and vice versa. When DRX occurs, deformation is easy to perform. Therefore, Q may be more suitable than lnZ to determine the critical deformation temperature and critical strain rate: 

Let:

(11) $$S={\left\{\frac{\partial \ln[\sinh(\alpha \sigma )]}{\partial \left(1/T\right)}\right\}}_{\dot{\varepsilon }}$$

**Figure 5:**
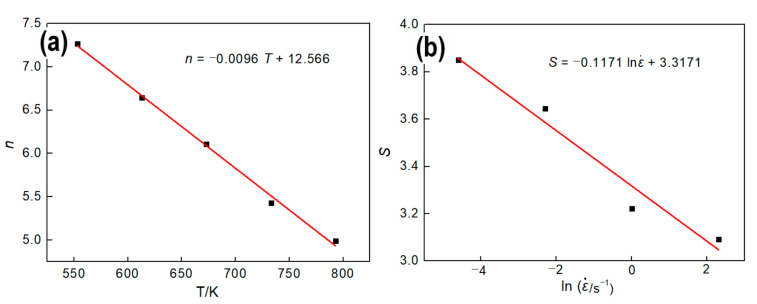
Relationship between activation energy and deformation parameters. (**a**) *T–n*, (**b**) $\ln\dot{\varepsilon }-S$

Then, Equation (10) can be expressed as:

(12) Q=RSn

where, *n* is the slope of the curve of $\ln(\dot{\varepsilon })$ to ln[sinh(ασ)] at different temperature as shown in Figure 4c, and *S* is the slope of the curve of ln[sinh(ασ)] to 1/T at various strain rate as shown in Figure 4d. Figure 5 shows the relationship between *n* and *T*, *S* and $\ln(\dot{\varepsilon })$. 

Obviously, *n* and *S* can be described as Equation (13) by linear fitting:

(13) $$\left\{ \begin{array}{l} S=-0.1171\times \ln\dot{\varepsilon }+3.3171\\ n=-0.0096\times T+12.566 \end{array}\right.$$

Substitute Equation (13) to Equation (12), *Q* can be written as Equation (14):

(14) $$Q=RSn=8.314\times (-0.1171\times \ln\dot{\varepsilon }+3.3171)\times (-0.0096\times T+12.566)$$

Equation (14) can be used to calculate the value of *Q* under the whole deformation conditions, and the results are shown in Table 3. Combining the microstructures of the samples and *Q* of each deformation conditions, we can find that DRX occurred in the samples when the value of *Q* is below 183.9 KJ/mol, and when *Q* is higher than 183.9 KJ/mol, only DRV takes place. Therefore, Q can be used to describe critical temperature and strain rate of DRX.

As shown in Table 3, Q value is significantly affected by deformation conditions, and taking the average Q to calculate lnZ under various deformation conditions will greatly affect the calculation accuracy. In order to obtain the exact value of lnZ, Equation (14) is substituted into Equation (1) and the calculation results are shown in Table 4. Obviously, the value of lnZ changed a lot compared with Table 2. As reported in [20,22] or in Table 2 of this paper, lnZ decreases with the increasing of temperature or decreasing of strain rate since the lnZ is calculated by average Q. However, lnZ in Table 4 of this paper decreases with the increasing of strain rate at 280~400℃ while increases at 460~520 °C when it is calculated by various Q. This indicates that in a wide deformation range, the calculation of lnZ by using average Q will not only affect the calculation results, but even fail to accurately reflect the variation rules of lnZ under different deformation conditions. Additionally, it can be found that lnZ is 35.5 under deformation condition of 340 °C/10s^−1^ in Table 4 and no DRX occurs in Table 1, but lnZ equals 30.04 at 340 °C/10 s^−1^ and DRX takes place in Table 1. This indicates again that lnZ is not suitable as the temperature and strain rate coupled DRX critical conditions for Al-4.8Mg alloy, even though the lnZ was calculated by various Q. Compared with pure aluminum in Ref [24], the added elements (Mg, Mn, Cr, etc.) of Al-4.8Mg alloy may have an great influence on DRX critical conditions of studied materials and change the critical conditions of dynamic recrystallization. This should be investigated in future research.

According to the above comparative analysis, it can be judged that the temperature and strain rate coupled DRX critical conditions of Al-4.8Mg alloy can be described by Q as Equation (15):

(15) $$\left\{ \begin{array}{l} Q=8.314\times (-0.1171\times \ln\dot{\varepsilon }\;+\;3.3171)\times (-0.0096\times T+12.566)\\ Q<183.9 \end{array}\right.$$

#### 3.3.2. Determination of Critical Strain

Equation (15) can be used to determine the critical temperature and strain rate of DRX occurrence. However, during the deformation process, even if the temperature and strain rate conform to Equation (15), DRX will occur in the samples only when the strain meets a critical value [20,22]. Generally, the work-hardening rate θ(θ=dσ/dε) was used to describe special point of stress-strain curve and determine the critical strain of DRX [21,22].

**Figure 6:**
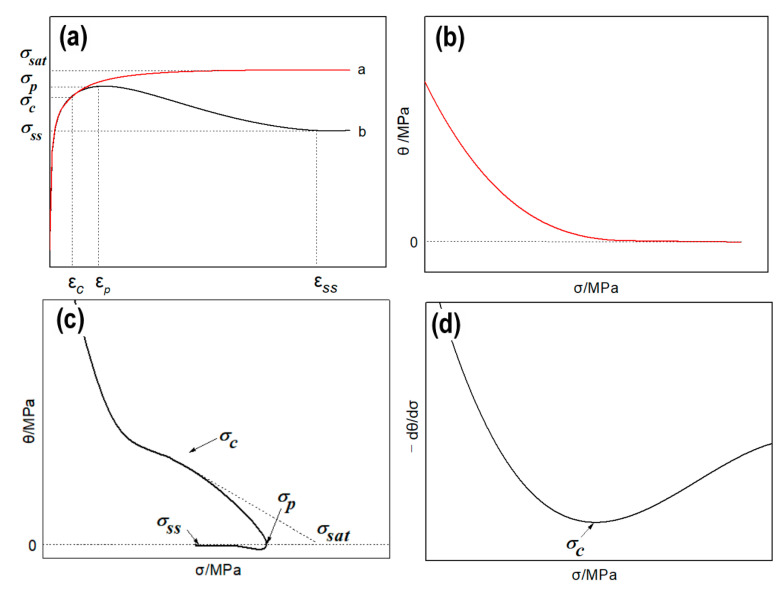
The relationship between strain hardening rate and flow stress. (**a**) σ-ε curve, (**b**) θ-σ curve for DRV, (**c**) θ-σ curve for DRX, (**d**) −∂θ/∂σ−σ curve for DRX.

As shown in Figure 6a, the stress curves can be divided into two different types. The ‘a’ curve is attributed to DRV dominated softening mechanism. The flow stress rapidly increases at the initial stage of the deformation due to the higher WH. When a balance between WH and DRV is reached, a saturation value ($\sigma_{sat}$) appears and remains constant. Therefore, as shown in the corresponding θ−σ curve (Figure 6b), θ gradually decreases to 0 with the increase of σ. The ‘b’ curve marked is considered that DRX is the main softening mechanism. In WH stage, the flow stress rapidly increases to a critical stress ($\sigma_{c}$) at the initial deformation stage due to the higher work-hardening. Once the strain exceeds the critical strain ($\varepsilon_{c}$), DRX occurs and the flow stress step into softening stage. The flow stress continues to slowly increase to the peak stress ($\sigma_{p}$), then begins to decrease until it reaches a steady stress ($\sigma_{ss}$). It can be seen from Figure 6c, after a sharp decline, θ decreases slowly until it reaches 0 ($\sigma_{p}$). With the continuous development of deformation, the strain decreases further to the lowest value and then slowly rises to 0 ($\sigma_{ss}$). The turning point on the curve before the peak stress is considered as the critical stress point of DRX ($\sigma_{c}$), which can be get from the lowest value of the −dθ/dσ−σ curve (Figure 6d), then the corresponding $\varepsilon_{c}$ can be got directly from stress-strain curves of Figure 2. Besides, the intersection between the tangent at the $\sigma_{c}$ and the abscissa is the $\sigma_{sat}$.

**Figure 7:**
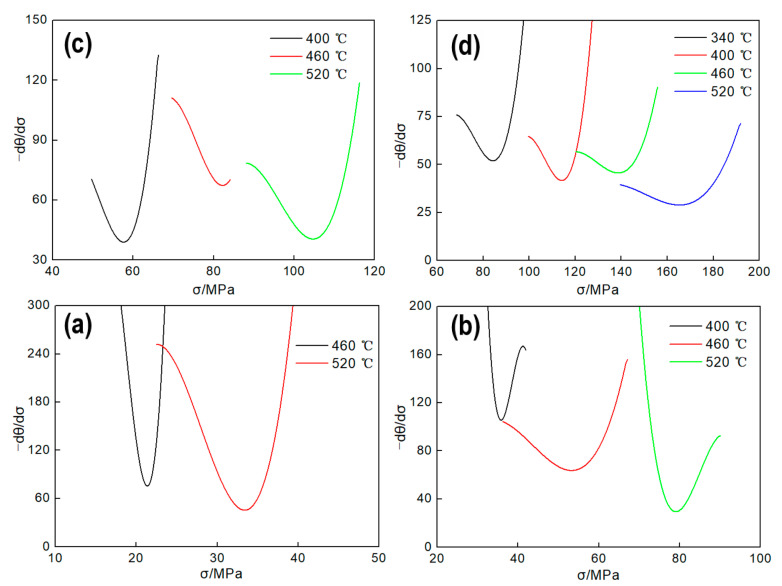
Relationship between strain hardening rate and flow stress of Al–4.8Mg alloy. (**a**) 0.01 s^−1^; (**b**) 0.1 s^−1^; (**c**) 1 s^−1^; (**d**) 10 s^−1^.

Based on the above analysis, the minimum value of the −dθ/dσ−σ curve under each deformation condition was calculated as shown in Figure 7. Thus, the corresponding critical strain of DRX can be obtained according to the flow stress curves shown of Figure 2, and the value of critical strain are shown in Table 5.

By analyzing the data in Table 5, $\varepsilon_{c}$ can be described by the temperature and strain rate, as Equation (16) shows. 

**Figure 8:**
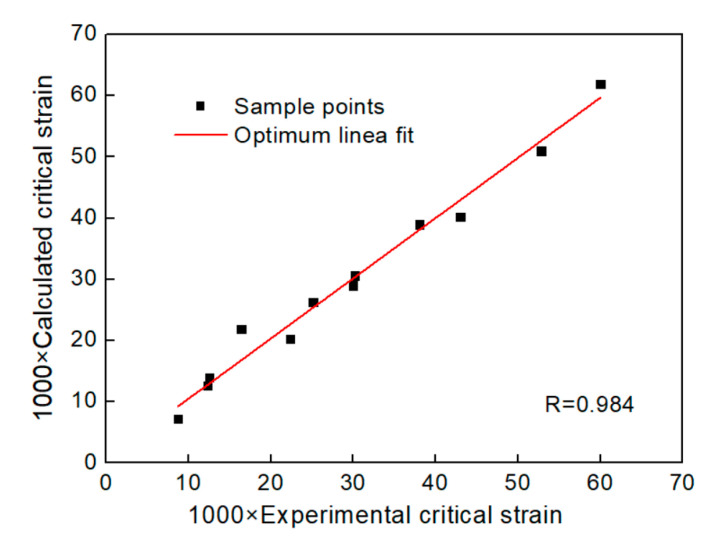
Correlation between the experimental critical strain and calculated critical strain.

The correlation coefficient between experimental value in Table 5 and calculated value by Equation (16) is 0.984 as shown in Figure 8, which shows that Equation (16) can accurately describe the critical strain of Al-4.8Mg alloy at various deformation conditions.

(16) $$1000\varepsilon_{c}=96.7633+16.5577\times \ln\dot{\varepsilon }-0.01477\times T-0.03682\times \ln\dot{\varepsilon }\times T-0.03682\times \ln{\dot{\varepsilon }}^{2}-0.0001\times T^{2}$$

Thus, the DRX critical conditions of sample under the whole deformation conditions in this paper can be established based on the deformation activation energy and work-hardening rate, as shown in Equation (17):

(17) $$\left\{ \begin{array}{l} Q=8.314\times (-0.1171\times \ln\dot{\varepsilon }\;+\;3.3171)\times (-0.0096\times T+12.566)\\ Q<183.9\\ 1000\varepsilon_{c}=96.7633+16.5577\times \ln\dot{\varepsilon }-0.01477\times T-0.03682\times \ln\dot{\varepsilon }\times T-0.03682\times \ln{\dot{\varepsilon }}^{2}-0.0001\times T^{2} \end{array}\right.$$

### 3.4. Physically–Based Constitutive Model of Flow Stress

In this paper, the dominating softening mechanism of the samples includes DRV, DRV+DRX and DRX. The critical conditions of DRX occurrence has been established in Section 3.3. Therefore, it can be confrimed that the samples, whose deformation behavior satisfies Equation (17), represents DRV+DRX or DRX dominant softening. Their constitutive model can be established based on the DRX model. While, for deformation behavior of the samples do not satisfy Equation (17), the constitutive model can be established based on the WH-DRV model. Therefore, in this paper, the constitutive model of the experimental samples should be segregated into two parts.

#### 3.4.1. Part I. Constitutive Model Based on WH-DRV

The flow stress of metals is mainly affected by the internal dislocation density, while the variation of dislocation density in the matrix depends on the generation and elimination speed of dislocations. Therefore, the relationship among dislocation density, WH, DRV and true strain can be described as Equation (18) [19]:

(18) $$\frac{d\rho }{d\varepsilon }=U-\Omega \rho$$

where, $\frac{d\rho }{d\varepsilon }$ is the rate of increase of the dislocation density, *U* is the WH, when the strain is defined in a certain value, it can be considered as a constant. Ωρ denotes the dislocation elimination and recombination caused by DRV, Ω is a coefficient of DRV. By integrating Equation (18), Equation (19) can be obtained [19]:

(19) $$\rho =\frac{U}{\Omega }-(\frac{U}{\Omega }-\rho_{0})e^{-\Omega \varepsilon }$$

where, $\rho_{0}$ is the initial dislocation density. Then the flow stress σ can be written as a function of ρ [19]

(20) $$\sigma =r\mu b\sqrt{\rho }\;$$

Therein, *r* is a material constant, *μ* is the shear modulus, *b* is the Burgers vector. Plugging Equation (19) in to Equation (20), the flow stress of DRX samples can be obtained, as described by Equation (21):

(21) $$\sigma ={\left[\sigma_{sat}^{2}+(\sigma_{0}^{2}-\sigma_{sat}^{2})e^{-\Omega \varepsilon }\right]}^{0.5}$$

where, $\sigma_{sat}$ is the saturation stress, and $\sigma_{0}$ is the yield stress. In order to establish the constitutive model of DRV, $\sigma_{sat}$, $\sigma_{0}$ and Ω need to be solved.

Saturation stress $\sigma_{sat}$ of the DRX samples can be obtained by the method shown in Figure 6. Considering the *θ*–σ curves of the DRV samples do not have inflection point, therefore, the peak stress of the DRV sample was approximated to their $\sigma_{sat}$. $\sigma_{0}$ can be obtained directly by Figure 2. Furthermore, by taking logarithm on both sides of Equation (21) and transforming it, we can get Equation (22):

(22) $$\Omega \varepsilon =\ln\left(\frac{\sigma_{sat}^{2}-\sigma_{0}^{2}}{\sigma_{sat}^{2}-\sigma^{2}}\right)$$

The DRV coefficient Ω can be obtained by substituting the typical parameters into Equation (22). For DRV samples, $0.9\sigma_{p}$ corresponding strain are substituted into Equation (22). For the deformation condition when DRX can take place, the critical stress and critical strain are substituted into Equation (22), and then the value of Ω under different deformation conditions can be obtained.

**Figure 9:**
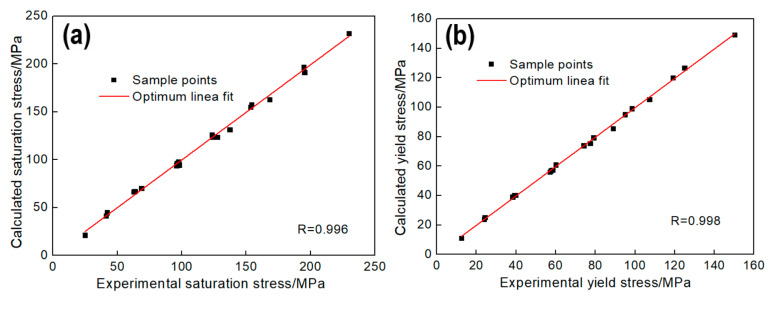

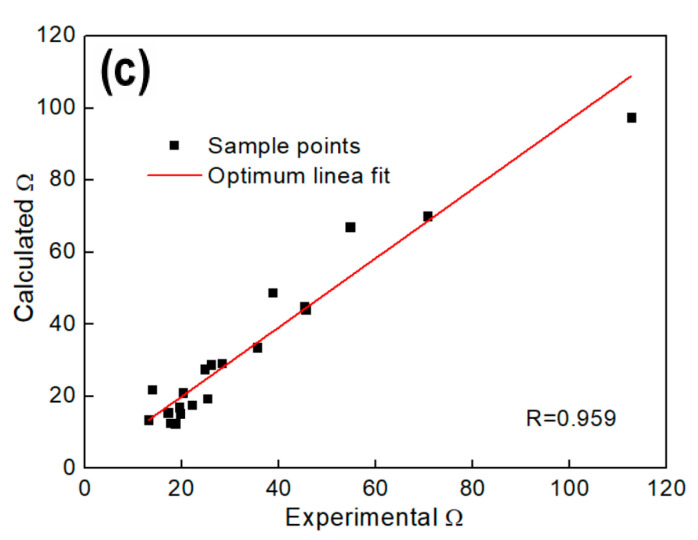
Correlation between the experimental values and calculated value: (**a**) $\sigma_{sat}$ (**b**) $\sigma_{0}$, (**c**) Ω.

It was found by regression analysis that $\sigma_{sat}$, $\sigma_{0}$ and Ω can be described by Equation (23), and the equation coefficients are shown in Table 6. As shown in Figure 9, there are good correlations between the test value and the calculated value:

(23) $$Y=E+F\times \ln\dot{\varepsilon }+G\times T+H\times \ln\dot{\varepsilon }\times T+I\times \ln{\dot{\varepsilon }}^{2}+J\times T^{2}$$

Thus, the constitutive model of the DRV stage of the experimental samples in the experimental range can be expressed as:

(24) $$\left\{ \begin{array}{l} \sigma ={\left[\sigma_{sat}^{2}+(\sigma_{0}^{2}-\sigma_{sat}^{2})e^{-\Omega \varepsilon }\right]}^{0.5}\\ \sigma_{sat}=788.26028+23.02247\times \ln\dot{\varepsilon }-1.44929\times T-0.01448\times \ln\dot{\varepsilon }\times T-0.18729\times \ln{\dot{\varepsilon }}^{2}+0.00069\times T^{2}\\ \sigma_{o}=568.86909+15.52259\times \ln\dot{\varepsilon }-1.10668\times T-0.01087\times \ln\dot{\varepsilon }\times T-0.12466\times \ln{\dot{\varepsilon }}^{2}+0.00056\times T^{2}\\ \Omega =399.36983+23.03059\times \ln\dot{\varepsilon }-1.27479\times T-0.0390\times \ln\dot{\varepsilon }\times T+0.5773\times \ln{\dot{\varepsilon }}^{2}+0.00105\times T^{2} \end{array}\right.$$

#### 3.4.2. Part II. Constitutive Model Based on DRX

When the deformation strain of samples satisfies Equation (17), DRX occurs in the samples, and represents a higher softening rate. Generally, the volume fraction of DRX grains can be described as Equation (25) [19]:

(25) $$X_{D}=1-\exp(-B{\left(\frac{t}{t_{x}}\right)}^{n})$$

where, *t* is the deformation time, $t_{x}$ is the time required to achieve a certain softening degree, and $\frac{t}{t_{x}}$ can be treated as a function related to nucleation rate and growth rate of DRX grains, Thus [19]:

(26) $$X_{D}=1-\exp(-k_{D}{\left(\frac{\varepsilon -\varepsilon_{c}}{\varepsilon_{p}}\right)}^{n_{D}})$$

where *k_D and_ n_D_* are the material constants, and generally, 0 < *k_D_* < 1 < 0 < *n_D_* ≤ 2. According to the published works [27], the volume fraction of DRX grains can be further expressed as Equation (27).

(27) $$X_{D}=\frac{\sigma_{rec}-\sigma }{\sigma_{sat}-\sigma_{ss}}$$

Therein, $\sigma_{rec}$ denotes the stress before occurrence of DRX, and it can be obtained from Equation (24). $\sigma_{sat}$ is the saturation stress, and $\sigma_{ss}$ is the steady stress. Thus, combining Equations (26) and (27), the flow stress of the samples in DRX state can be expressed as Equation (28) [19]:

(28) $$\sigma =\sigma_{rec}-(\sigma_{sat}-\sigma_{ss})\{1-\exp{\left[-k_{d}(\frac{\varepsilon -\varepsilon_{c}}{\varepsilon_{p}})\right]}^{n_{d}}\}$$

**Figure 10:**
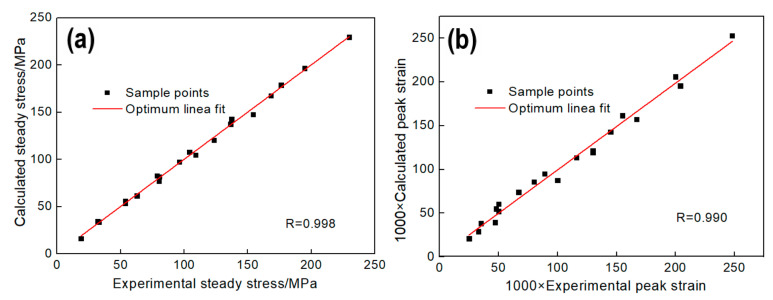
Correlation between the experimental values and calculated value: (**a**) $\sigma_{ss}$, (**b**) $\varepsilon_{p}$.

In order to calculate the parameters of the model, the steady stress $\sigma_{ss}$ and the peak strain $\varepsilon_{p}$ should be obtained, as well as the material constants $n_{D}$, $k_{D}$. Steady stress $\sigma_{ss}$ can be calculated by the method shown in Figure 6 and peak strain $\varepsilon_{p}$ can be got directly by Figure 2. $\sigma_{ss}$ and $\varepsilon_{p}$ can be described by Equation (23), and the equation coefficients are shown in Table 7. As shown in Figure 10, it exhibits good correlations between the test value and the calculated value.

**Figure 11:**
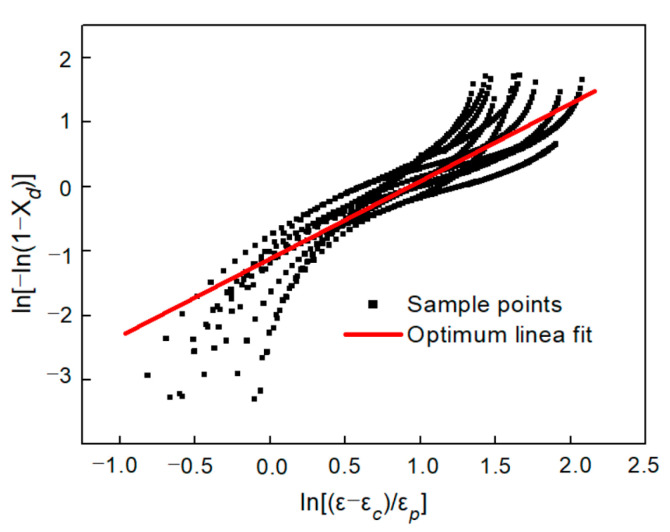
Relation between DRX volume fraction and strain.

Combining Equations (26) and (27), Equation (29) can be obtained. Take logarithm on both sides of Equation (29) to obtain Equation (30). Thus, $n_{D}$ and $k_{D}$ can be obtained by calculating the slope and intercept of $\ln[-\ln(1-X_{d})]$ to $\ln(\frac{\varepsilon -\varepsilon_{c}}{\varepsilon_{p}})$ curve, as shown in Figure 11. Therefore, the constitutive model of the flow stress in DRX state can be expressed as Equation (31):

(29) $$X_{D}=\frac{\sigma_{rec}-\sigma }{\sigma_{sat}-\sigma_{ss}}=1-\exp(-k_{d}{\left(\frac{\varepsilon -\varepsilon_{c}}{\varepsilon_{p}}\right)}^{n_{d}})$$

(30) $$\ln[-\ln(1-X_{d})]=n_{d}\ln(\frac{\varepsilon -\varepsilon_{c}}{\varepsilon_{p}})+\ln k_{d}$$

(31) $$\left\{ \begin{array}{l} \sigma =\sigma_{rec}-(\sigma_{sat}-\sigma_{ss})\{1-\exp{\left[-0.331(\frac{\varepsilon -\varepsilon_{c}}{\varepsilon_{p}})\right]}^{1.246}\}\\ \sigma_{sat}=788.26028+23.02247\times \ln\dot{\varepsilon }-1.44929\times T-0.01448\times \ln\dot{\varepsilon }\times T-0.18729\times \ln{\dot{\varepsilon }}^{2}+0.00069\times T^{2}\\ \sigma_{ss}=879.62333+53.84443\times \ln\dot{\varepsilon }-1.58280\times T-0.06159\times \ln\dot{\varepsilon }\times T-0.21229\times \ln{\dot{\varepsilon }}^{2}+0.00066\times T^{2}\\ 1000\varepsilon_{c}=96.7633+16.5577\times \ln\dot{\varepsilon }-0.01477\times T-0.03682\times \ln\dot{\varepsilon }\times T-0.03682\times \ln{\dot{\varepsilon }}^{2}-0.0001\times T^{2}\\ 1000\varepsilon_{p}=1073.28708+20.54150\times \ln\dot{\varepsilon }-2.28161\times T-0.01285\times \ln\dot{\varepsilon }\times T-0.38822\times \ln{\dot{\varepsilon }}^{2}+0.00126\times T^{2} \end{array}\right.$$

#### 3.4.3. Verification of the Constitutive Model

**Figure 12:**
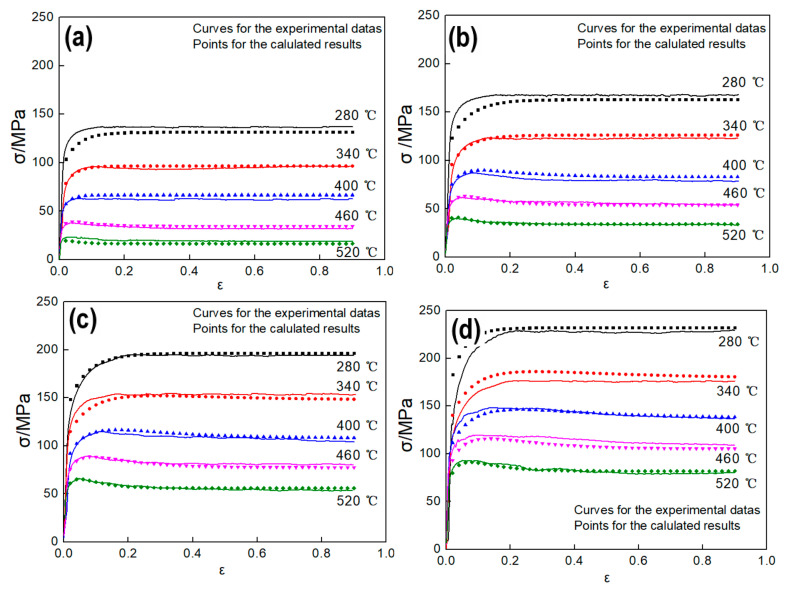
Comparison between the experimental and predicted flow stress curves. (**a**) 0.01 s^−1^, (**b**) 0.1 s^−1^, (**c**) 1 s^−1^, (**d**) 10 s^−1^.

To verify the model, first, Equation (17) was applied to determine the softening mechanisms of the samples under the verifying deformation condition. Then, the parameters of the verifying deformation conditions in DRV state were substituted to Equation (24), and correspondingly, the parameters in DRX state were substituted to Equation (31). At last, the obtained predicted flow stress value was compared with the experimental flow stress. Figure 12 shows the comparison of calculated and experimental flow stress. Obviously, the predicted flow stresses are highly consistent with the experimental flow stress.

**Figure 13:**
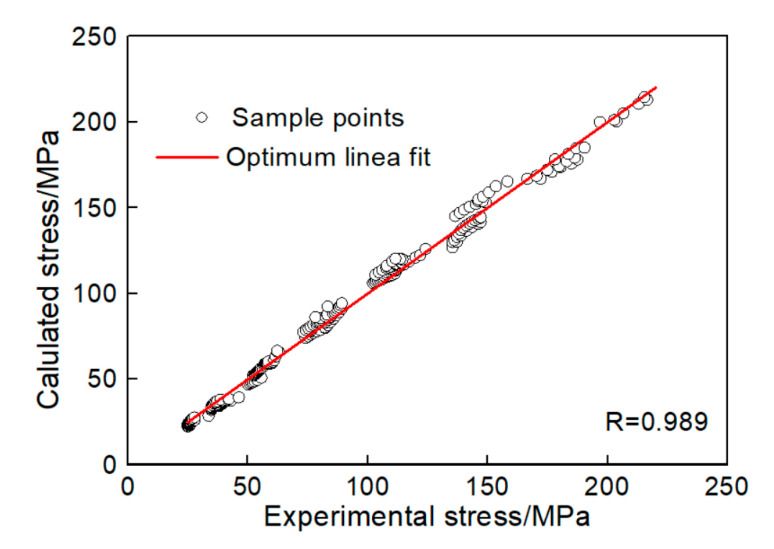
Correlation between the experimental value and calculated value.

To further verify the model, the correlation between the experimental value and calculated value are shown in Figure 13, and the correlation coefficient (*R*) and the average absolute relative error (*AARE*) between the calculated flow stress and experimental flow stress were obtained though Equations (32) and (33) [8]:

(32) $$R=\frac{\sum \begin{array}{l} N\\ i=1 \end{array}(\sigma_{E}^{i}-{\bar{\sigma }}_{E})(\sigma_{C}^{i}-{\bar{\sigma }}_{C})}{\sqrt{\sum \begin{array}{l} N\\ i=1 \end{array}{\left(\sigma_{E}^{i}-{\bar{\sigma }}_{E}\right)}^{2}}\sqrt{\sum \begin{array}{l} N\\ i=1 \end{array}{\left(\sigma_{C}^{i}-{\bar{\sigma }}_{C}\right)}^{2}}}$$

(33) $$AARE(\%)=\frac{1}{N}\sum_{i=1}^{N}|\frac{\sigma_{E}^{i}-\sigma_{C}^{i}}{\sigma_{C}^{i}}|\times 100\%$$

where, $\sigma_{E}^{i}$ is the experimental flow stress, ${\bar{\sigma }}_{E}$ is mean value of $\sigma_{E}^{i}$; $\sigma_{C}^{i}$ is the calculated flow stress, ${\bar{\sigma }}_{C}$ is its mean value of $\sigma_{C}^{i}$, and *N* is the number of samples. By calculating, the correlation coefficient R is 98.9%, and *AARE* is 4.02%. This indicates the established constitutive model in this paper is of high accuracy.

## 4. Conclusions

In this study, the flow behavior of Al–4.8Mg alloy was investigated via thermo–mechanical tests in a range of deformation temperatures (280–520 °C) and strain rates (0.01–10 s^−1^). The main conclusions can be summarized as follows:

(1) There are three main dynamic softening mechanisms of samples within the range of experimental parameters: DRV, DRV + DRX and DRX, and the equiaxial DRX is more easily to form under higher deformation temperature and higher strain rate.
(2) Based on the deformation activation energy and work-hardening rate, an improved DRX critical conditions model was proposed to describe the DRX conditions of Al-4.8Mg alloy under a wide range of hot deformation parameters.
(3) According to the proposed DRX critical conditions model, a two–stage physically–based constitutive model including the WH-DRV stage and DRX stage was established. The 4.02% of AARE between the calculated values and experimental values indicates that the established physically–based constitutive model has a good prediction accuracy.

## Figures and Tables

**Table 1:** Optical microstructure of the Al–4.8Mg alloy under various deformation conditions.

Deformation Parameter	280 °C	340 °C	400 °C	460 °C	520 °C
0.01 s^–1^	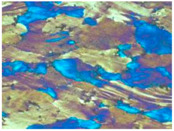	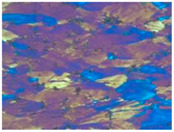	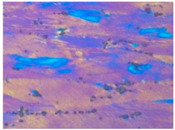	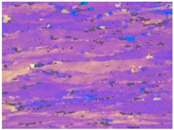	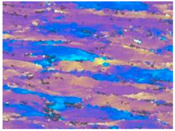
0.1 s^–1^	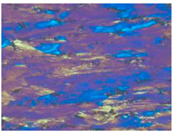	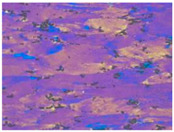	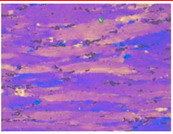	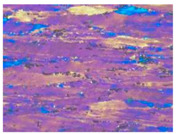	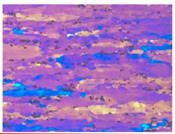
1 s^–1^	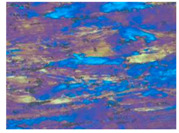	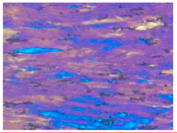	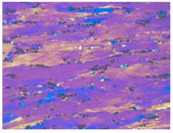	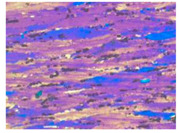	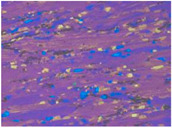
10 s^–1^	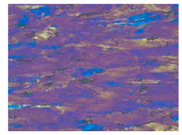	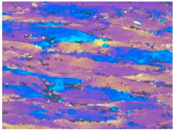	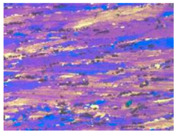	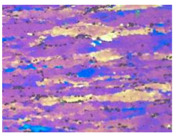	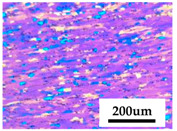
DRV		DRX			DRX + DRV

**Table 2:** The lnZ of Al–4.8Mg alloy under various deformation conditions calculated by average Q.

Deformation Parameter	280 °C	340 °C	400 °C	460 °C	520 °C
0.01 s^–1^	33.4	29.7	26.6	24.1	21.9
0.1 s^–1^	35.7	32.0	28.9	26.4	24.2
1 s^–1^	38.0	34.3	31.2	28.7	26.5
10 s^–1^	40.3	36.6	33.5	31.0	28.8

**Table 3:** The deformation activity energy at different hot deformation conditions.

Deformation Parameter	280 °C	340 °C	400 °C	460 °C	520 °C
0.01 s^–1^	232.7	214.2	195.7	183.4	158.8
0.1 s^–1^	216.4	199.2	182.1	170.6	147.7
1 s^–1^	200.1	184.3	168.4	157.8	136.6
10 s^–1^	183.9	169.3	154.7	145.0	125.5

**Table 4:** The lnZ of Al–4.8Mg alloy under various deformation conditions calculated by various Q.

Deformation Parameter	280 °C	340 °C	400 °C	460 °C	520 °C
0.01 s^–1^	46.0	37.4	30.4	24.5	19.5
0.1 s^–1^	44.8	36.8	30.2	24.8	20.1
1 s^–1^	43.5	36.2	30.1	25.0	20.7
10 s^–1^	42.3	35.5	29.9	25.3	21.3

**Table 5:** The critical strain of Al–4.8Mg alloy under different hot deformation conditions.

Deformation Parameter	280 °C	340 °C	400 °C	460 °C	520 °C
0.01 s^–1^	/	/	/	0.0123	0.0087
0.1 s^–1^	/	/	0.0300	0.0164	0.0125
1 s^–1^	/	/	0.0430	0.0302	0.0223
10 s^–1^	/	0.0600	0.0528	0.0380	0.0251

**Table 6:** The coefficients of Equation (23) about $\sigma_{sat}$, $\sigma_{0}$ and Ω.

*Y*	*E*	*F*	*G*	*H*	*I*	*J*
$\sigma_{sat}$	788.26028	23.02247	−1.44929	−0.01448	0.18729	0.00069
$\sigma_{0}$	568.86909	15.52260	−1.10668	−0.01087	0.12466	0.00056
Ω	399.36983	23.03059	−1.27480	−0.03902	0.57730	0.00105

**Table 7:** The coefficients of Equation (23) about $\sigma_{ss}$ and $\varepsilon_{p}$.

*Y*	*E*	*F*	*G*	*H*	*I*	*J*
$\sigma_{ss}$	879.62333	53.84443	−1.58280	−0.06159	0.21229	0.00066
$\varepsilon_{p}$	1073.28708	20.54150	−2.28161	−0.01285	0.38822	0.00126
